# An Unsentimental Appeal

**Published:** 1921-02-12

**Authors:** 


### An Unsentimental Appeal.

In the study of disease by the laymen of neighbouring countries the People's League of Health is carrying through an important experiment. The aim is to send instructed Englishmen and Englishwomen to France, Switzerland, and other countries in order that they may learn there how the great problems of national regeneration are being dealt with. To this end Sir German Sims Woodhead, the distinguished Professor of Pathology at Cambridge University, together with other medical authorities, have consented to deliver a series of thirteen weekly instructive and educational lectures in London on the care of the body, the first of which was given last Wednesday. The course, at Morley Hall, George Street, Hanover Square, W., will conclude with an optional examination for certificates issued by the Medical Council of the League, with travelling scholarship prizes consisting of a week's visit to a foreign country, with facilities for visiting its health centres.

The League recently issued an appeal to the public for funds to further its valuable work, which is to increase public information and education to such an extent that no mother shall lose her child through ignorance, that no man or woman shall be stricken with disease for lack of knowledge.

